# Modulation of antimicrobial efflux pumps of the major facilitator superfamily in *Staphylococcus aureus*

**DOI:** 10.3934/microbiol.2018.1.1

**Published:** 2018-01-04

**Authors:** Manjusha Lekshmi, Parvathi Ammini, Jones Adjei, Leslie M. Sanford, Ugina Shrestha, Sanath Kumar, Manuel F. Varela

**Affiliations:** 1 QC Laboratory, Harvest and Post Harvest Technology Division, ICAR-Central Institute of Fisheries Education (CIFE), Seven Bungalows, Versova, Andheri (W), Mumbai, 400061, India; 2 CSIR-National Institute of Oceanography (NIO), Regional Centre, Dr. Salim Ali Road, Kochi, 682018, India; 3 Eastern New Mexico, Department of Biology, Station 33, 1500 South Avenue K, Portales, NM, 88130, USA

**Keywords:** *Staphylococcus aureus*, efflux pump inhibitors, modulation, multidrug resistance, antimicrobial resistance, bacteria, pathogens

## Abstract

Variants of the microorganism *Staphylococcus aureus* which are resistant to antimicrobial agents exist as causative agents of serious infectious disease and constitute a considerable public health concern. One of the main antimicrobial resistance mechanisms harbored by *S. aureus* pathogens is exemplified by integral membrane transport systems that actively remove antimicrobial agents from bacteria where the cytoplasmic drug targets reside, thus allowing the bacteria to survive and grow. An important class of solute transporter proteins, called the major facilitator superfamily, includes related and homologous passive and secondary active transport systems, many of which are antimicrobial efflux pumps. Transporters of the major facilitator superfamily, which confer antimicrobial efflux and bacterial resistance in *S. aureus*, are good targets for development of resistance-modifying agents, such as efflux pump inhibition. Such modulatory action upon these antimicrobial efflux systems of the major facilitator superfamily in *S. aureus* may circumvent resistance and restore the clinical efficacy of therapy towards *S. aureus* infection.

## 1. Introduction

**Figure 1.**
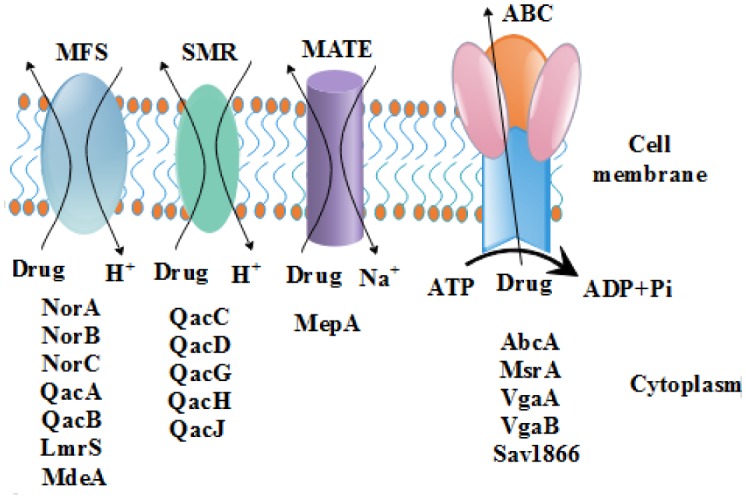
Efflux pumps of *Staphylococcus aureus* belonging to the MFS, SMR, MATE and ABC families of membrane proteins. Except the ABC group of efflux pumps which use hydrolysis of ATP to energize the drug transport, all other efflux proteins depend on the electrochemical gradient created by ions for energization of their drug transport activities.

Bacteria are capable of overcoming the accumulation of drugs by transporting them to the outer membrane by a mechanism of active efflux which is mediated by drug efflux pumps. These drug efflux pumps can be grouped into primary and secondary active transporters. The primary active transporter proteins consist of pumps which utilize energy in a form of ATP to transport drugs across the membrane by a mechanism of ATP hydrolysis [1],[2]. On the contrary, the secondary active transporters encompass those membrane proteins that use energy from a concentration gradient formerly established by a primary active transport process to transport a solute across the cellular membrane [3]. They indirectly use the energy derived from ATP hydrolysis and transport molecules across an electrochemical concentration gradient by coupling with another compound [4]. Here, the driving forces are H^+^ or Na^+^, transported down the concentration gradient with the substrate being carried simultaneously against this concentration gradient (Figure 1).

Secondary active membrane transporters are highly substrate specific, and are involved in the transport of substances like peptides, sugars, vitamins, fatty acids, amino acids, neurotransmitters, etc. [5]. The substrate specific solute recognition sites on these transporters are used as antimicrobial drug targets. There are two types of secondary active transport namely (i) symport, where the driving force ion and the substrate are transported in the same direction, and (ii) antiport, where both the ion and substrate are transported in opposite directions.

Bacterial secondary active transporters involved in antimicrobial drug mechanisms are grouped under four families based on their sequence and functional similarities, namely:

(i) Major facilitator super family (MFS);
(ii) Resistance-nodulation-cell division transporter super family (RND);
(iii) Small multidrug resistant transporter family (SMR);
(iv) Multiple antimicrobial extrusion protein family (MATE).

The first three families include H^+^/drug antiporters, whereas the last one includes H^+^/drug and Na^+^/drug antiporters [6]. Staphylococcal efflux pumps belong to the MFS, SMR and MATE transporter families. Interestingly, a new RND pump, FarE, from *S. aureus* was identified and predicted to transport arachidonic and linoleic acids to confer fatty acid resistance [7],[8].

In order to study these and other solute transporters, the general methodology involves cloning of the efflux pump gene and its expression in an antibiotic hypersensitive *E. coli* such as the KAM32 of Tsuchiya and colleagues [9]. However, in the host strain, the expression of the efflux gene may be under the control of a transcriptional regulator or communication signals such as those involved in quorum sensing [10]. The prevailing hypothesis is that the actual function of efflux pump could be the extrusion of toxic metabolites, Krebs cycle intermediates, quorum sensing signals and other unknown molecules and the transport of antimicrobials is incidental perhaps because they resemble the actual substrates of efflux pumps [11]. Gene knockout and comparative transcriptome analysis of mutant versus the wild type can help to identify the functions of efflux pumps and their essential nature to the host bacterium.

## 2. The major facilitator superfamily (MFS)

The MFS represents an ancient, diverse, and largest family of secondary active transporters conserved from bacteria to humans, with over 10,000 sequenced members. They are ubiquitously found in all three kingdoms of living organisms. The MFS protein transporters target a broad variety of substrates including ions, carbohydrates, lipids, amino acids and peptides, nucleosides, antibiotics and other molecules [12],[13]. They constitute many efflux pumps comprising of uniporters, symporters and antiporters involved in antibacterial drug resistance in bacteria [14]–[19]. These efflux pumps are located on the cytoplasmic or plasma membrane of bacteria preventing drug accumulation inside bacterial cells thereby conferring drug resistance. The MFS is grouped further into 76 subfamilies based on phylogenetic analysis, substrate specificity and operational mechanism, in the Transporter Classification Database (TCDB) [19]. The MFS efflux proteins can be divided into two distinct clusters with either 12 or 14 transmembrane segments. The efflux proteins of MFS are of the antiporter group and genes encoding these efflux pumps are mostly chromosomal, but some are plasmid borne.

## 3. Other families of solute transporters

The SMR family consists of small, hydrophobic bacterial multidrug resistance efflux proteins that function as homo-oligomeric complexes [20]. The proteins of SMR family transport only antibiotics and lipophilic compounds [20],[21]. SMR consists of 18 recognized subfamilies, each with a characteristic function. The efflux pumps Smr, QacG, QacH and QacJ with plasmid-encoded genes in *S. aureus* confer resistance to ethidium bromide and quaternary ammonium compounds [22],[23]. The efflux pump encoded by the chromosomal gene *mepA* is the first and the only multidrug transporter from the MATE family to be reported in *S. aureus*. It confers low-level resistance to quaternary ammonium compounds, antibiotics such as ciprofloxacin, norfloxacin and the dyes [24].

RND and ABC efflux proteins are multicomponent and the genes encoding these domains may be present in an operon or in different locations on the genome [25],[26]. RND efflux pumps such as the well-studied MexAB-OprM of *Pseudomonas aeruginosa* and AcrAB-TolC of *Escherichia coli* are tripartite systems [26],[27]. In AcrAB-TolC tripartite complex, AcrB RND protein complexes with a periplasmic membrane fusion protein (AcrA) and an outermmbrane protein (OMP) (TolC) [28],[29].

## 4. Staphylococcal efflux pumps of the major facilitator superfamily (MFS)

Gram-positive organisms such as *S. aureus*, *Streptococcus* species, *Enterococcus* species etc resistant to multiple drugs can complicate the chemotherapy. Over a period, these bacteria have colonized the hospital environment and because of their constant exposure to antibiotics and harsh chemicals such as disinfectants and sanitizers, these bacteria have gained enormous resistance. Nosocomial infections involving such bacteria have become a formidable problem being responsible for high morbidities and mortalities. One of the major reasons for the antimicrobial resistance of these bacteria is efflux pumps.

Efflux pumps are key modulators of antimicrobial resistance in *S. aureus* and the critical roles they play in the antibiotic resistance has been established with the identification of efflux substrates of several efflux pumps. The genome of *S. aureus* has more than 30 putative efflux pumps [30]. The whole genome sequences of pathogens available in the public databases offer many opportunities to identify and characterize the efflux pumps of unknown function. LmrS of S. *aureus* and EmrD3 of *Vibrio cholerae* were identified by genome scanning, followed by cloning and substrate identification [31],[32]. This approach is relatively simple in the case of efflux pumps such as those belonging to MFS family which are single proteins involved in efflux activity.

In *S. aureus*, the drug: H^+^ antiporter-2 (DHA-2) subfamily is of great significance. In addition to drugs, the DHA-2 family members also transport bile salts and dyes [19]. Among the efflux pumps of the MFS family, QacA/B efflux pumps, belonging to the 14 TMS cluster, are some of the earliest ones discovered from *S. aureus* that resist biocides such as quaternary ammonium compounds, other antiseptic and disinfectant substances. NorA is one of the most studied MFS efflux pumps of *S. aureus* [33]. NorB and NorC, the efflux pumps structurally similar to NorA, belong to the 12 TMS cluster [34]. Other important MFS efflux pumps of *S. aureus* include MdeA and LmrS [18],[32]. Efflux pumps of *S. aureus* belonging to the MFS family that have been identified and characterized with respect to their predicted structure, substrate profiles and contribution to antimicrobial resistance, are shown in Table 1. The genes encoding these efflux pumps may be present in the chromosome or carried on the plasmids. The following sections describe structural and functional aspects of staphylococcal efflux pumps of MFS family.

**Table 1.** Staphylococcal efflux pumps of MFS family and their substrates.

Efflux pump	Location of the coding genes	Substrates	Reference
NorA	Chromosome	Fluoroquinolones, biocides and dyes	[33]–[37]
NorB	Chromosome	Fluoroquinolones, tetracycline, biocides and dyes	[34],[38],[39]
NorC	Chromosome	Fluoroquinolones	[40]
QacA	Plasmid	Quaternary ammonium compounds, guanyl hydrazones, biguanidines, diamidines	[41]–[43]
QacB	Plasmid	Quaternary ammonium compounds, tetraphenylphosphonium, ethidium bromide, acriflavine, rhodamine	[40],[42]
SdrM	Chromosome	Norfloxacin, biocides and dyes	[44]
MdeA	Chromosome	Ciprofloxacin, macrolides	[23]
LmrS	Chromosome	Linezolid, chloramphenicol, florfenicol, trimethoprim, erythromycin, kanamycin, fusidic acid, lincomycin, Streptomycin, tetraphenylphosphonium, ethidium bromide	[32]
Tet38	Chromosome	Tetracyclines	[45]
TetA(K)	Plasmid	Tetracyclines	[46]–[48]

### 4.1. NorA, NorB and NorC efflux proteins

NorA was the first efflux pump to be discovered in a fluoroquinolone resistant *S. aureus* [34],[35]. NorA, together with QacA and QacB, constitute the best-characterized efflux pumps from *S. aureus* [18],[22]. NorA has 388 amino acids that fold into 12 transmembrane helices with a molecular weight of 42.2 kDa. NorA is chromosomally encoded and shares 40% amino acid identity with Bmr efflux pump of *Bacillus subtilis* [24]. It was initially thought that fluoroquinolones were the only substrates of NorA efflux pump, but later works showed that NorA extrudes an array of non-fluoroquinolone antibiotics such as tetracycline and an array of chemicals including ethidium bromide, cetrimide and tetraphenylphosphonium (TPP) [16]. The substrate diversity of NorA-mediated efflux is exemplified by the fact that various quinolones like norfloxacin and ciprofloxacin are transported by it [22]. The expression of *norA* is negatively regulated by MgrA [33]. Inactivation of MgrA resulted in enhanced expression of the *norA* gene and elevated resistance to fluoroquinolones and other antimicrobials, and depending upon the genetic nature of the background strain, the MgrA regulator protein may act either as a repressor or an activator of *norA* gene expression [33]. Exposure of *S. aureus* to compounds such as ethidium bromide can have modulatory effect on the antimicrobial resistance through the activities of efflux pumps. Couto, et al. reported deletion of a 70 bp region in NorA promoter that resulted in a 35-fold increase in the expression of *norA* and decreased susceptibility of the strain to quinolones and biocides [49].

The polypeptide chains of NorB and NorC are made up 464 and 462 amino acids, respectively [33],[40]. NorB and NorC have about 61% amino acid identity, although structurally NorB has 12 transmembrane segments while NorC has 14 segments [38]. NorB has 30% sequence similarity with NorA and Bmr efflux pumps [40]. Unlike NorA, both NorB and NorC confer resistance to both hydrophilic quinolones such as ciprofloxacin, norfloxacin, and hydrophobic quinolones, like moxifloxacin, and their activities are negatively regulated by MgrA [33],[40]. Overexpression of both *norC* and *norB* in an *mgrA* mutant strain results in a quinolone-resistant phenotypic mutant in a mouse subcutaneous abscess model, and the expression of NorB was upregulated, and the increased expression positively correlated with the expression of MgrA [38],[40]. Deletion of the *norB* gene resulted in compromised growth and survival of the mutant strain. These observations strongly suggest a key role for NorB in the virulence and fitness of *S. aureus*. Little is known about the NorD efflux pump except that it does not efflux substrates of the Nor group of efflux pumps and that its expression is upregulated at pH 5.5 [38],[40].

### 4.2. Qac group of efflux pumps

The Qac group of efflux pumps consists of QacA, QacB, QacG, QacH and QacJ transporters. Of these, the QacA and QacB efflux pumps remain the best characterized efflux pumps found in *S. aureus* bacteria [50]. QacA and QacB are MFS transporters, both 514 amino acids long and 55 kDa in size, and assume similar 2-D predicted structures with 14 TMS [50]–[52]. QacA and QacB are different from each other by seven amino acid residues [41]. An acidic amino acid in TMS10 is critical for the binding of divalent cations [41]. An important feature of these efflux pumps is that they are plasmid-borne making them transmissible to those bacteria which may require them to tide over antimicrobial pressure and survive better. While QacA is encoded on pSK1 plasmid, QacB is found on pSK23 [41]. Horizontal acquisition of efflux pumps result in the creation of a pool of bacteria, which were previously sensitive to disinfectants, but not anymore. QacA/B efflux pumps have been reported in many bacteria and in all these, the genes have been acquired through HGT. Quaternary ammonium compunds, guanyl hydrazones, biguanidines, diamidines and a wide range of dyes form substrates of QacA [51]. QacB differs from QacA in that it is not able to efflux divalent cationic compounds, diamidines, and biguanidines. Considering the broad substrate range of QacA compared to QacB hypothesis suggesting the evolution of QacA from QacB has been proposed [51]. The expressions of *qacA* and *qacB* are regulated by the *qacR* regulator belonging to the *tetR* family of transcriptional regulators, in a substrate-depended manner [52].

### 4.3. TetA(K) and Tet38 efflux pumps

TetA(K), first reported in a strain of *S. aureus*, has 459 amino acids that form 12 transmembrane segments [46]–[48]. The TetA(K) efflux pump is characterized by its ability to confer high resistance to the tetracycline class of antibiotics.

Tet38 is a chromosomally encoded MFS efflux pump that confers resistance to tetracyclines and some fatty acids and contributes to bacterial colonization of skin and survival in abscess environment [45],[53]. The fatty acid substrates of Tet38 include linoleic, palmitoleic, and undecanoic acids, but not palmitic acid and polyamines [53]. Tet38 is a 19 kDa protein with 450 amino acids that form 14 transmembrane helices, sharing 26% amino acid similarity with TetA(K) [54],[55].

### 4.4. SdrM efflux pump

The SdrM drug transporter is responsible for enhanced resistance to antimicrobial agents such as norfloxacin, acriflavine and ethidium bromide in a MRSA strain of *S. aureus* N315 [23]. With 447 amino acids and 14 predicted transmembrane segments, SdrM has 68% and 65% amino acid sequence similarities with NorB and QacA, respectively [44]. Unlike most other efflux pumps, ethidium bromide was only a moderate substrate for SdrM and instead, acriflavin was a good substrate [44]. Based on the putative MgrA-binding promoter sequence, a possible MgrA-depended expression of SdrM has been proposed [44].

### 4.5. MdeA efflux pump

The MdeA protein is a member of the MFS family of efflux pumps made up of 479 amino acids that form 14 transmembrane helices [56]. This 52 kDa protein effluxes fluoroquinolones, although with low affinity [57]. Efflux with low levels of sequence similarity with MdeA include QacA (23% similarity), EmrB of *E. coli*, LmrB of *B. subtilis* and FarB of *Neisseria gonorrhoeae* [58]. Mutations in promoter region of MdeA result in the overexpression of the pump [59].

## 5. Modulation of and transcriptional regulation of antimicrobial efflux pumps from the major facilitator superfamily

This section deals primarily with modulation of drug efflux pump activity, which involves mainly inhibition of substrate efflux activities from the antimicrobial transporters. The gene expression programs of the genetic determinants that encode antimicrobial resistance may be altered in several instances. These latter cases involve regulation at the level of transcription.

### 5.1. QacA and QacB modulation

One of the first plasmid encoded multidrug efflux pump systems of the major facilitator superfamily from *S. aureus* to be characterized, QacA is also one of the first such pumps to be observed in which ethidium efflux was inhibited by the oxidative phosphorylation uncoupler, carbonyl cyanide *m*-chlorophenyl hydrazone, indicating that QacA is a proton-driven drug pump and thus a secondary active antimicrobial transporter [60],[61]. A follow-up study showed that the transport of additional QacA substrates 4′,6-diamidino-2-phenylindole (DAPI), 3′,3′-dipropyloxacarbocyanine (DiOC_3_), and pyronin Y, were all inhibited by carbonyl cyanide *m*-chlorophenyl hydrazone, further confirming that the *S. aureus* QacA drug efflux pump system is driven by the proton motive force [62]. The same study showed that uncouplers reserpine and verapamil, and ionophores valinomycin and nigericin effectively inhibited ethidium transport across the membrane through QacA, indicating an involvement of both the membrane potential and the pH gradient in mediating antimicrobial efflux by QacA [62].

Like its closely related counterpart QacA above, efflux pump activity of the plasmid encoded QacB was inhibited by carbonyl cyanide *m*-chlorophenyl hydrazone [63] and possibly reserpine [64]. As of this writing, however, no other modulators have been studied in QacB from *S. aureus*.

### 5.2. TetA(K) modulation

Although the TetA(K), Tet(K), or TetK tetracycline efflux pump from *S. aureus* is related to but not considered a true multidrug efflux pump, Tet(K) nonetheless has functions other than tetracycline efflux, such as ion exchange and substrate-metal complex transport [47], and it may thus be more promiscuous than previously thought. Tet(K) is also a good target for modulation [47]. One of the first reports of Tet(K) modulation showed that the pump was inhibited by the siderophore called nocardamine [65]. Early work also showed that the semi-synthetic tetracycline analog called 13-cyclopentylthio-5-OH-tetracycline (13-CPTC) inhibited the growth of host cells harboring the Tet(K) efflux pump [66]. The bioactive agent epigallocatechin-gallate from extracts of green tea inhibited tetracycline transport mediated by Tet(K) to a certain extent in *Staphylococcus epidermidis* host cells [67]. In recent studies, a series of compounds derived from coumarin showed lowered tetracycline MIC levels in *S. aureus* expressing the Tet(K) tetracycline efflux pump system [68]. Interestingly, the vitamin D_3_ agent cholecalciferol, but not the vitamin E agent α-tocopherol, reduced the tetracycline MIC in *S. aureus* IS-58 containing the Tet(K) efflux pump [69]. Remarkably, biofilm development was inhibited by two phytochemicals, 7-hydroxycourmarin and indole-3-carbinol, in *S. aureus* cells that overexpress Tet(K) [70]. Recent work showed that a bioactive component α-terpinene, a plant derived essential oil from *Chenopodium ambrosioides* inhibited growth of strain IS-58 harboring Tet(K) [71]. Other compounds that are present in essential oils from the plant *Salvia officinalis* and *S. sclarea* inhibited the efflux pump activity of Tet(K) [72].

### 5.3. Tet38 regulation

Expression of the Tet38 tetracycline efflux pump has been demonstrated to be negatively regulated at the level of transcription by MgrA, a global regulator, by an indirect means [34]. A more recent study has shown that the *tet38* gene is also regulated by the so-called tetracycline regulator 21 (TetR21) in *S. aureus* [45]. As far as we know, no modulators (inhibitors) of Tet38 efflux activity have been reported.

### 5.4. NorA modulation

Of the many antimicrobial efflux pump systems of the major facilitator superfamily harbored by *S. aureus*, NorA is the most widely studied in terms of efflux pump inhibition, and modulation of the multidrug efflux pump NorA has recently been extensively reviewed elsewhere [18],[23],[73]–[79]. NorA represents an excellent experimental model system for the development and evaluation of efflux pump inhibitors [77],[79]. Therefore, this section will briefly summarize recent reports pertaining to NorA efflux pump modulation.

In 2016, a three-dimensional model of NorA from *S. aureus* was generated using homology modeling based on the known crystal structure of the glycerol-3-phosphate transport protein from *E. coli* [80],[81]. The resulting homology modelled NorA structure was then embedded in the hydrophobic biological membrane using molecular dynamics simulations, and then docked to known inhibitors and substrates [81]. Next, the NorA structure was screened *in silico* for binding by new molecules in order to retrieve novel putative efflux pump inhibitors [81]. These investigators found that the overall structural NorA model includes a putative substrate binding cleft consisting of hydrophobic residues Val-44 (helix 2), Phe-47 (helix 2), Gln-51 (helix 2), Phe-140 (helix 5), Ile-244 (helix 8), Gly-248 (helix 8) and Phe-303 (helix 10) [81]. These residues are conserved in transporters of the major facilitator superfamily [13],[16],[74],[75],[82],[83]. In particular, residues that are present in helix 5 of the major facilitator superfamily efflux pumps compose the so-called antiporter motif or Motif C [43],[84],[85], which has been shown to be mechanistically functional and to work in a hinge-like manner with residues of helix 8 of related antimicrobial efflux transporters [86],[87].

Starting in 2014, a group of synthetic heterocyclic boronic acid derivatives were shown to be effective inhibitors of NorA-driven accumulations studied involving ethidium bromide [88]. One of these compounds in particular, 6-benzyloxypyridine-3-boronic acid, was more recently used as a platform upon which to synthesize new species, denoted as 3i and 3j, both of which showed enhanced growth inhibitory activities on *S. aureus* [89]. The 3i and 3j species further showed enhanced inhibitory effects on NorA-mediated accumulation of ethidium bromide [89].

In 2015, based on a study showing potentiation by pyrrole alkaloid derivatives of antimicrobial activities by ciprofloxacin and ethidium bromide in *S. aureus* with NorA [90], a naturally occurring plant root compound and P-glycoprotein inhibitor, boeravinone B, from *Boerhavia diffusa*, was found to inhibit growth of *S. aureus* and ethidium transport by NorA, in addition to reducing biofilm formation [91].

When tested for ethidium efflux activity, chemical variations that were introduced to a dithiazole thione backbone produced a derivative DTT10 that showed indirect inhibition of ethidium efflux through other pumps from *S. aureus*, such as NorB or MepA, but at higher concentrations the inhibitor demonstrated a competitive inhibition with substrate for binding to NorA [92]. Terpenes and terpenoids, like the oxygenated monoterpenes called nerol and 3,7-dimethyl-octan-1-ol, that are derived from plant essential oils, inhibit ethidium transport across the membrane in an inducible *S. aureus* strain that overproduces NorA [93]. A synthetic variant of the fruit-based riparin compound called Riparin-B was demonstrated to effectively inhibit ethidium efflux in another *S. aureus* strain that overproduces NorA [94]. Tannic acid, a polyphenol compound found in plants as an intermediate metabolite, was reported to diminish the MIC of ethidium and norfloxacin in host cells containing NorA, implicating the tannin compound as a putative efflux pump inhibitor [95]. Amide derivatives of piperic acid and 4-ethylpiperic acid effectively inhibited ethidium efflux by NorA and reduced the MICs for ciprofloxacin in host cells, one of which overexpresses NorA [96]. Using benzothiazine as a core platform for synthesis of derivatives, a new compound in the series, 2-phenylquinoline 6c, showed good efflux pump inhibition of ethidium transport by NorA [97]. More recently, a racemic comparison of the (R)-versus the (S)-enantomeric versions of 2-phenylquinoline showed that the (R)-enantiomer had better efflux pump inhibitory activity in NorA [98].

Using ethidium MIC assays, one recent study showed indirect inhibition of NorA by a variety of flavonoid compounds hesperetin, phloretin, diosmetin, myricitrin and quercitrin, compounds of which were extracted from fruits like apples [99]. A study that focused on the indole scaffold moiety as the bioactive site of action for efflux pump inhibitors identified four new derivatives with good activities against efflux by NorA [100]. A more recent follow-up study showed that these flavonoids inhibited biofilm formation composed of host cells harboring NorA [101], and a similar result was previously observed in *S. aureus* host cells harboring NorA or the class K tetracycline efflux pump and inhibition of biofilm formation using two phytochemical agents 7-hydroxycoumarin and indole-3-carbinol [70]. Most recently, it was shown in cells harboring *norA* and other resistance determinants *norB*, *norC*, *mdeA*, *sdrM*, etc., that gene expression was down-regulated by phenolic blueberry and blackberry pomace extracts, resulting in enhanced susceptibilities to multiple antimicrobial agents; although efflux via Nor was not directly measured in the study [102]. A promising new *in silico* molecular modelling study involving so-called 3D pharmacophore model development has identified potentially new efflux pump inhibitors, called ModB and ModC, of NorA [103]. Along similar lines, using rational design methodology, new indole-based efflux inhibitors were identified as demonstrating potent activities against ethidium bromide efflux by NorA [104]. Taken together, approaches like these, as applied towards the modulation of multidrug efflux may potentially be of tremendous importance in clinical settings.

### 5.5. Modulation of additional S. aureus MFS pumps: MdeA, SdrM, Mef(A), FexA, and LmrS

Ethidium transport by the MdeA multidrug efflux pump from S. aureus was inhibited by piperine; and the combination of piperine and MdeA substrate mupirocin showed a synergistic effect in terms of inhibiting S. aureus growth [105]. Exposure of S. aureus strains to low amounts of various antimicrobial agents resulted in upregulation of determinants norA, norC and mdeA indicating the presence of alternative modes of modulation as mediated by changes in gene expression and regulation [106]. Thus, we predict that the MdeA multidrug efflux pump will be an important modulation target.

Inhibition of acriflavine efflux was observed in the presence of the oxidative phosphorylation uncoupler carbonyl cyanide *m*-chlorophenyl hydrazone in the SdrM multidrug efflux pump from *S. aureus* indicating that the pump is driven by a proton gradient and is therefore a secondary active transporter [44]. To date, however, no other modulators of efflux activity has been reported for this newer SdrM multidrug efflux pump [44].

As far as we are aware, the Mef(A) macrolide efflux pump and distant member of the major facilitator superfamily [107] has not yet been subjected to inhibitory or other modulatory studies in *S. aureus*; although the determinant is present in clinical isolates [108]. It may be interesting to evaluate this particular efflux system in *S. aureus*.

A mobile resistance determinant encoding an efflux pump for chloramphenicol and florfenicol FexA [109] was found in several agriculture isolates [110]. However, no modulators for FexA have thus far been reported in the literature.

In the *S. aureus* LmrS multidrug efflux pump, ethidium efflux inhibition was observed with carbonyl cyanide *m*-chlorophenyl hydrazone [32]. On the other hand, reserpine did not inhibit drug efflux suggesting that it is a substrate for LmrS [32]. More recently, we showed that ethidium transport by LmrS was inhibited by cumin extract from the spice plant *Cuminum cyminum*, and we found that at low cumin concentrations a direct effect on LmrS was suggested while at higher cumin concentrations an indirect effect on LmrS, possibly through a collapse of the respiratory chain, was found [111]. Most recently, it was found that the TetR21 transcriptional regulator influences LmrS gene expression, and since TetR21 did not bind to the *lmrS* promoter, it is suggested that regulation of LmrS occurs by an indirect mode [112]. Speculatively, these results imply that one or more of the LmrS substrates may interact somehow with the TetR21 system to mediate resistance.

## 6. Future directions

In addition to finding new and safe modulators for antimicrobial efflux pumps of the major facilitator superfamily present in *S. aureus*, it will become increasingly important to understand the physiological mechanisms inherent during antimicrobial efflux across the membrane by these related transporters. A detailed understanding of the basic antimicrobial translocation cycle through an efflux pump will be beneficial in efficiently designing new putative modulators.

Furthermore, it remains unknown how the various known modulators manage to disrupt the antimicrobial efflux mechanisms through the transporters; towards this, it is poorly understood whether the modulators function to inhibit drug efflux through competitive binding with substrate, through allosteric sites, or indirectly through disturbance of the energizing mode that mediates coupling of antimicrobial efflux with ion translocation through the pumps or through disruption of the ion gradient energy. What is further unclear is how the antimicrobial transporters dictate whether one substrate, a small few substrates or many multiple substrates are determined. Along these lines, it is poorly understood how these antimicrobial efflux pumps permit their larger substrate molecules to be transported while keeping smaller molecules, such as water and ions tightly impermeable.

Future work also entails gaining a true understanding of how established and new modulators can be applied to clinical practice in terms of safety, effective dosages, mode of administration to patients, and synergistic combinations between a modulator and clinically important antimicrobial agents or between two distinctive modulators. Clinical investigators will be interested in acquiring listings of the infectious diseases that may be amenable to modulation. Lastly, it will be of interest to pursue both synthetic and naturally occurring modulators.
